# A comparative analysis of root dentin transparency with known age

**DOI:** 10.4103/0974-2948.71052

**Published:** 2010

**Authors:** Anita Singhal, V Ramesh, PD Balamurali

**Affiliations:** *Department of Oral Pathology and Microbiology, H.P. Government Dental College and Hospital, Shimla - 171 001, India*; 1 *Mahatma Gandhi Post Graduate Institute Of Dental Sciences, Puducherry - 605 006, India*

**Keywords:** Age estimation, dentin transparency, gustafson’s criteria

## Abstract

**Objective:**

To correlate dimensions of root transparency and age, and to assess whether transparency is reliable for age estimation of unknown.

**Materials and Methods:**

50 freshly extracted single rooted permanent teeth from 50 different individuals (27 males and 23 females) were collected and their ground sections of 400 μm were stained with 1% methylene blue. The area of the translucent zone was measured by superimposing a transparent graph paper on the ground section under stereomicroscope. The length of the translucency was measured by using digital vernier caliper.

**Results:**

A strong positive correlation between age and translucency of dentin was noted. The length rather than the area of the translucent zone correlated more with age.

**Conclusion:**

Translucency of the root dentin increases with age and it can be used as a reliable parameter for the age estimation.

## Introduction

Age estimation to establish the identity of a person for ethical, humanitarian, and official records, particularly in the legal and criminal investigations in the field of forensic science, is of paramount importance. Various modalities are available in the assessment of age of a person such as skeletal and dental changes. Age estimation by dentition is an important subspecialty of forensic sciences. Age estimation is also important for living individuals whose chronological age is under dispute. Dental age is one of the few measures of physiological development that are uniformly applicable from infancy to late adolescence. After attaining maturity, teeth continue to undergo changes making age estimation possible among adults.[1] Gustafson considered six factors for estimation of age. One of the most reliable of Gustafson’s criteria for age estimation is by measurement of root dentin transparency.[2] Transparency starts in the apical part of root and increases with age in the coronal direction.[3] This particular change is least affected by environmental factors and the pathological process.[2,4,5] It also shows symmetrical distribution on both sides of jaws.[6]

The present study was done to find the total root dentin transparency including area and length in ground sections of 50 freshly extracted teeth, to correlate the measured transparency with the known age of the individual and to evaluate the reliability of root transparency for age estimation.

## Materials and Methods

**Figure 1:**
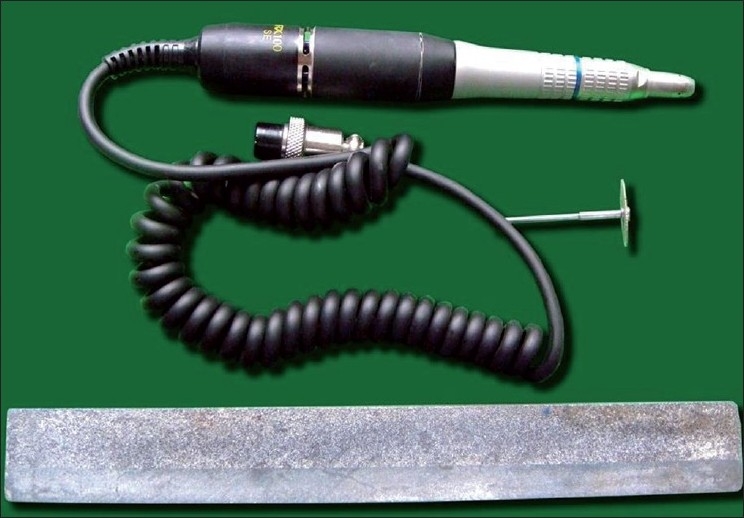
Armamentarium for the preparation of the ground section of a tooth

**Figure 2:**
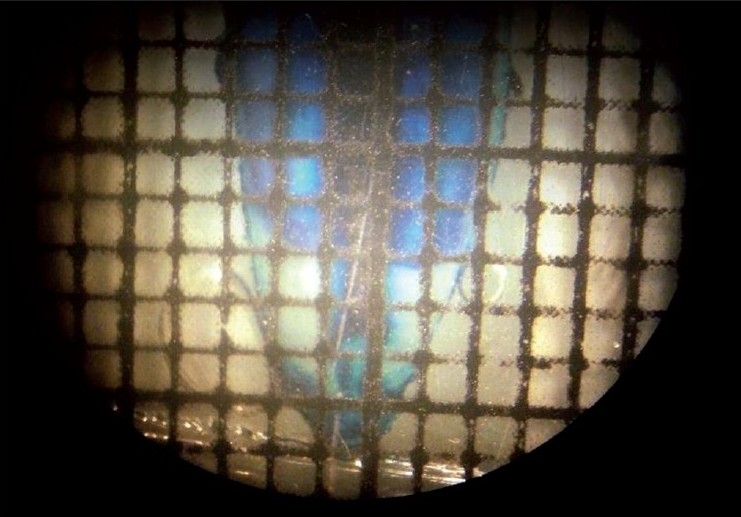
Ground section under a stereomicroscope with a graph paper superimposed

Fifty freshly extracted permanent lower central incisors from 50 different individuals were collected from the Department of Oral and Maxillofacial Surgery, Mahatma Gandhi Post Graduate Institute of Dental Sciences, Puducherry. There were 27 male and 23 female individuals with their age ranging from 27 years to 77 years. Permanent teeth extracted for obvious clinical reasons such as periodontal problems, orthodontic purposes, and prosthodontic purposes were included for the study. Teeth with root caries, abrasion, erosion, and external resorption were excluded from the study. Buccolingual sections of the tooth were made up to the thickness of 400 μm by using micromotor and Arkansas stone [Figure 1]. The thickness of sections was measured by using digital screw gauge. The ground section was then stained with 1% methylene blue. The whole ground section was stained but sclerosed dentin remained unstained. The area and length of both the translucency and root were measured. In order to measure the length and area of the root, a horizontal line was drawn with a pencil along the CEJ on the ground section. The ground section was then observed under stereomicroscope to measure the area. Over the ground section, transparent graph paper was superimposed [Figure 2]. The number of squares was counted in the total root area, and in the translucent apical zone. Counting was done according to the following method: one completely filled square was taken as 1 mm^2^, more than half filled square was also taken as 1 mm^2^, and less than half filled square was not counted.

**Figure 3:**
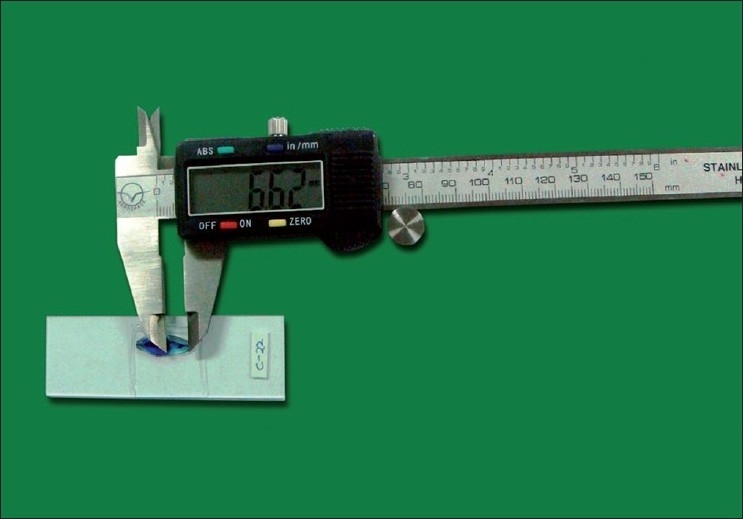
Length determination of the translucent zone using digital vernier caliper

The total number of squares in the total root area gave the area of the root, and in the translucent zone the area of the translucent zone. The ratio of translucent zone to total root area was calculated. For measuring the length of the root and translucent zone, the ground section was kept against a continuous fixed light source. The total length of the root and translucent zone length were measured by using digital vernier caliper [Figure 3]. The length of the apical translucent zone was measured separately on the buccal side and lingual side. Averages of both sides were taken. The ratio of this translucent zone to total root length was calculated.

### Statistical analysis

The statistical analysis was carried out using Statistical Package for Social Sciences (SPSS Inc., Chicago, IL, version 13.0 for Windows). Mean, median, and standard deviation were calculated for area and length values separately. Pearson’s correlation coefficient was calculated to see the correlation between length and age, then area and age. Regression analysis was done to find the estimated age from length and area separately. The paired *t*-test was applied to see the difference between actual age and estimated age. Also Pearson’s correlation coefficient was calculated for actual and estimated age. All statistical tests were two-sided and performed at a significance level of α= 0.01.

## Results

The mean age of the sample was 51.57 years, median being 55 years. The standard deviation was 15.68 years and the standard error being 2.24.

Quantitative analysis of the ratio of translucent zone and root length measurements in ground sections revealed that the mean of the ratio is 0.38 and median is 0.40. Minimum and maximum ratios derived are 0.07 and 0.74 with a standard deviation of 0.16.

The mean ratio for the translucent zone area was 0.25 with a median value of 0.21. Minimum and maximum ratios observed are 0.02 and 0.60. The standard deviation observed is 0.14.

### Regression analysis results (length values)

- Y-intercept—22.25; it is the lowest value of age at which translucency of the dentin was zero.
- Coefficient of slope—77.04 is a measure of change in translucency when age changes by unit. (That is when age changes by a year; there is a 0.77 mm change in the translucency length).
- Correlation coefficient (r)—0.81 denotes the correlation between age and length of translucency.
- Coefficient of determination (r^2^ =0.657)–66% is a proportion of the variability explained by the regression equation.

Formula derived for age estimation:

Age=22.25 + 77.04 × ratio (C.I)(C.S)

**Figure 4:**
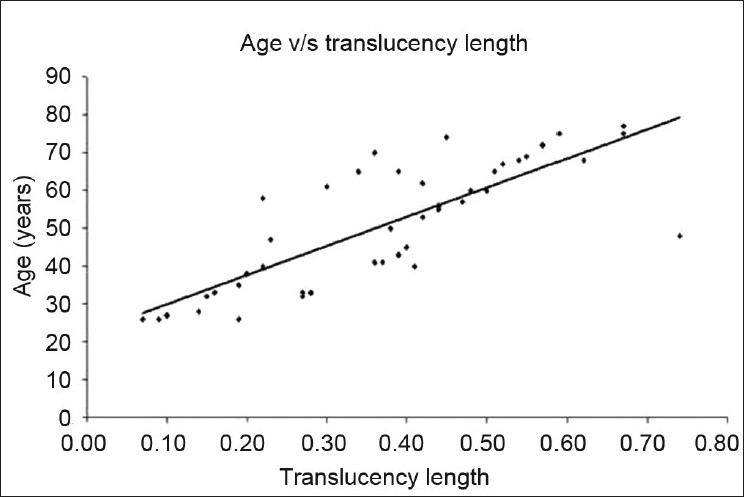
Scatter-plot showing age – translucent zone (length) association

It is seen from the Figure 4 that most of the cases fall near the midline.

### Regression analysis results (area values)

- Y-intercept—31.42 (t=9.54; *P*<0.0001) is the lowest value of age at which translucency of the dentin was zero.
- Coefficient of slope—79.51 (t=6.88; *P*<0.0001) is a measure of change in translucency when age changes by unit. (That is when age changes by a year there is a 0.79 mm^2^ change in the translucency area).
- Coefficient of correlation—0.70 denotes the correlation between age and area of translucency.
- Coefficient of determination—0.50 is a proportion of the variability explained by the regression equation.

Formula for estimation of age:

Age=31.42 + 79.51 × ratio (C.I)(C.S)

**Figure 5:**
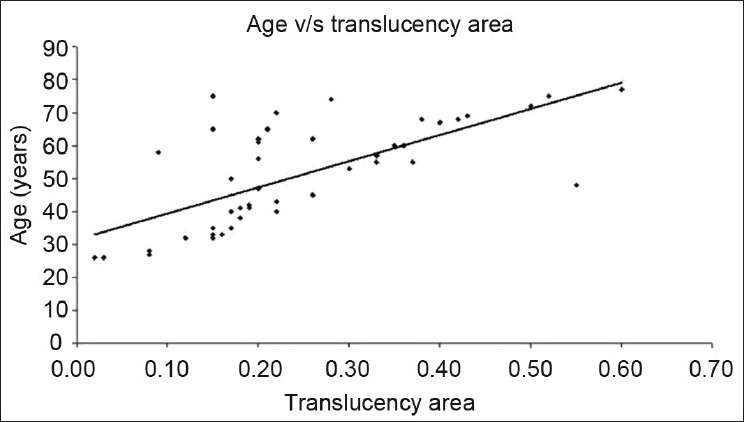
Scatter-plot showing the age – translucent zone (area) correlation

It is seen from the Figure 5 that most of the cases deviate away from the midline.

## Discussion

Age estimation by measuring transparent dentin is one of the Gustafson’s six criteria. Transparent dentin has been studied in the intact tooth in order to correlate with the age of the person as done by earlier reports of Solheim.[6] However, this is a three-dimensional phenomenon which encounters difficulties in the measurement procedures. Alternatively transparent dentin can also be studied in different thickness of sectioned tooth as has been done by Miles,[7] Bang and Ramm[3] Whittaker and Embry.[8] To be more accurate in the measurement of transparent dentin, the procedure of dye imbibitions was adopted by using 1% methylene blue.[8] The sclerosed dentin remained colourless in this method, normal dentin stained blue, and cementum took up dark blue colour. This was possible because the transparent dentin is made up of completely mineralized tubules that did not permit the entry of dye into this zone. This procedure was adopted by Whittaker and Bakri.[9] The translucency of dentin as noted in the ground section is due to an increase in intratubular mineralization. This increase in mineralization has same refractive index as that of peritubular dentin giving translucent appearance within dentin.[10] This translucency is first noted in the apical part of the tooth because of lesser diameter of dentinal tubules in the root dentin compared to the coronal part. Also lesser number of dentinal tubules are noted per unit area in apical part as stated by Nalabandian.[4] The increase in translucency is generally considered as a physiological change with aging process as proved by Azaz *et al*.,[5] in his study of impacted canines. They reported increase in dentin translucency with increasing age, even in impacted tooth which is away from any pathological and functional stimuli.

In the present study, there is a strong correlation between the translucencies with advancement of age which is supported by previous reports.[6,9,11] It is interesting to note that there is a gradual and definitive increase in the ratio of this translucency (length as a parameter) with that of increase in age. This was particularly prominent when noted in different decades of age. The correlation coefficient obtained in the study was 0.81 which was similar to that obtained in previous studies.[5,7,11] It was found that with increase in the age by 1 year, the length of translucency in the root also increases by 0.77 mm as depicted by the coefficient of slope. A further scatter plot was drawn against age and length of translucency. It was found that many variables were falling near the midline. The correlation coefficient obtained in this study by using area as a parameter was 0.70 which is almost similar to the figure achieved by Johnson (r= 0.71)[11] and Vasiliadis (r=0.86).[12] Forevery 1 year increase in age, there is an increase of 0.80 mm ^2^ in the translucent area in the apical part of dentin. Further the scatter diagram was plotted against age and translucent zone ratio (area) which clearly signifies that only few values fell close to midline as compared to the length of translucency.

However, in some of the teeth in this study, younger individuals have shown more translucent zone, which resulted in overestimation of age. This flaw may be attributed to the presence of periodontal infections and diseases of the pulp. The chronic periodontal infection may stimulate far more mineralization resulting in the increased translucent zone in the root part of dentin. Similarly, there was a contradictory factor that a lesser amount of translucency was encountered in some old individuals in our study that resulted in underestimation of age. This is probably due to slowing down of the process of sclerotic dentin formation in some of the individuals for various reasons. Further, beyond the age group, it is also possible that increase in the translucency does not take place, because it could have attained the highest level by blocking all the dentinal tubules in that area and thus giving a static value of translucency after a particular age. In a study done using Lamendin aging method on skeletal samples that were interred in the 18th and 19th centuries, more post-mortem changes were seen such as complete loss of root translucency, compared to the present study where freshly extracted teeth were used, and no case was seen with complete loss of root translucency. Thirty-five percent of their sample showed no root translucency, indicating that caution is required when applying the Lamendin method to archaeological or historical remains.[13]

If the parameter of translucent length and area of tooth is compared, the length shows more reliability as compared with translucent area of the tooth. The correlation coefficient of length of translucency is 0.81 compared to the correlation coefficient of area of translucency that is 0.70, which means 81% of cases are positively correlated in the age assessment utilizing the length of translucency compared to area. Furthermore, the coefficient of determination was 66% when the length of translucency was taken into consideration, in contrast the coefficient of determination was only 50% when area was used. The results of this study correlate with the previous studies.[6,8,10,14] Thomas *et al*.,[3] further suggested that dentin translucency using linear vernier measurement is more reliable than area plotting.

## Summary and Conclusion

This study in general concludes that translucency noted in the apical part of the tooth can be used for estimating the age of an individual. The two variables compared are length and area of the translucency. Statistical analysis showed that the translucency length is more reliable and accuracy of age estimation is greater. This method can be adopted in the age estimation of an individual using the formula (i) by length and (ii) by area. However, after the age of 70 years there seems to be a static point in dentinal translucency, which may be due to complete blockage of all dentinal tubules, beyond which it may be difficult to find the age of a person unless more number of cases are taken up for the study.
